# Resveratrol and its derivative pterostilbene ameliorate intestine injury in intrauterine growth-retarded weanling piglets by modulating redox status and gut microbiota

**DOI:** 10.1186/s40104-021-00589-9

**Published:** 2021-06-10

**Authors:** Yanan Chen, Hao Zhang, Yueping Chen, Peilu Jia, Shuli Ji, Yuying Zhang, Tian Wang

**Affiliations:** College of Animal Science and Technology, Nanjing Agricultural University, No.1 Weigang Street, Nanjing, Jiangsu 210095 People’s Republic of China

**Keywords:** Gut microbiota, Intestinal injury, Intrauterine growth retardation, Oxidative stress, Piglets, Pterostilbene, Resveratrol

## Abstract

**Background:**

Intestinal disorder is an important factor contributing to growth lag and high rates of morbidity and mortality of piglets with intrauterine growth retardation (IUGR). Resveratrol (RSV) and its derivative pterostilbene (PT) are natural stilbenes possessing various bioactivities, such as antioxidative and anti-inflammatory effects. This study compared the protective potential of RSV and PT on the intestinal redox status and gut microbiota in weanling piglets with IUGR.

**Methods:**

Eighteen male piglets of normal body weight (NBW) and 54 same-sex IUGR piglets were chosen according to their birth and weaning weights. The NBW piglets accepted a basal diet, while the IUGR piglets were allotted to one of three groups according to their body weight at weaning and received a basal diet, an RSV-supplemented diet (300 mg/kg), or a PT-supplemented diet (300 mg/kg), respectively.

**Results:**

Compared with IUGR piglets, both RSV and PT improved the IUGR-associated decrease in jejunal villus height and increases in plasma diamine oxidase activity and D-lactate level and jejunal apoptosis of piglets (*P* < 0.05). Administering RSV and PT also enhanced jejunal superoxide dismutase activity and the mRNA and protein expression of superoxide dismutase 2 of IUGR piglets by promoting nuclear factor erythroid 2-related factor 2 (Nrf2) nuclear translocation (*P* < 0.05). Comparatively, PT was more effective than RSV in elevating the villus height/crypt depth ratio and occludin mRNA and protein levels in the jejunum of IUGR piglets (*P* < 0.05). PT was also superior to RSV in increasing Nrf2 nuclear translocation and inhibiting malondialdehyde accumulation in the jejunum of IUGR piglets (*P* < 0.05). Additionally, RSV modulated the composition of cecal microbiota of IUGR piglets, as evidenced by increasing the prevalence of the phylum Bacteroidetes and the genera *Prevotella*, *Faecalibacterium,* and *Parabacteroides* and inhibiting the growth of the phylum Proteobacteria and its genera *Escherichia* and *Actinobacillus* (*P* < 0.05). Moreover, RSV significantly increased the butyrate concentration in the cecum of IUGR piglets (*P* < 0.05).

**Conclusion:**

PT is more potent than RSV to prevent intestinal oxidative stress, while RSV has a stronger capacity to regulate gut microbiota compared to PT.

**Supplementary Information:**

The online version contains supplementary material available at 10.1186/s40104-021-00589-9.

## Background

Maternal malnutrition and placental insufficiency perturb the growth and development of the embryo/fetus and its organs during pregnancy and induce a low birth weight, which is defined as intrauterine growth retardation (IUGR) [1]. IUGR not only delays postnatal growth but also incurs developmental deficits in multiple organ systems and high rates of postnatal mortality and morbidity, inflicting a great burden on livestock production and animal health [2]. The intestine, an essential apparatus driving the digestion, absorption, and metabolism of nutrients, is susceptible to multiple cellular or environmental stress responses owing to its continuous exposure to foreign substances, such as microorganisms, antigens, and toxins [3]. Accumulating evidence documents that IUGR leads to impaired intestinal development, oxidative stress, barrier dysfunction, and gut microbiota dysbiosis, thereby posing a high risk of intestinal disorders [4–7].

Oxidative stress is considered to be a trigger for the onset and development of intestinal diseases and it is implicated in the pathophysiology of IUGR-associated intestinal injury [7, 8]. At birth, the fetus suffers an abrupt transition from a hypoxic intrauterine environment with a low presence of free radicals to a relatively hyperoxic extrauterine environment, which generates a great amount of reactive oxygen and provokes oxidative aggression in several organs, including the intestine [9]. Notably, this process takes place during a time when the intestinal antioxidant defense mechanisms are still underdeveloped, particularly in IUGR neonates, making it difficult to tackle reactive oxygen overproduction and resultant oxidative damage [7, 9]. Our previous work demonstrated that intestinal oxidative injury persistently occurred in the small intestine of IUGR piglets from the neonatal to fattening stages [7, 10, 11]. Moreover, oxidative stress stimulates intestinal tight junction proteins to dissociate from the cytoskeleton and increases intestinal permeability, which facilities the passage of bacteria and endotoxins across the intestinal epithelium barrier and into the systemic circulation, causing further damage to multiple tissues and organ [12]. Accordingly, fortifying the intestinal antioxidative system of IUGR animals with appropriate antioxidants may be a promising approach for preventing or treating IUGR-associated diseases.

Resveratrol (RSV) is a natural stilbene, mainly present in red wine, grapes, rhubarb, and blueberries, conferring multiple biological benefits, including anti-inflammation, anticancer, and lipid/glucose metabolism-regulating effects [13]. Recent work shows that RSV has therapeutic potential for intestinal oxidative injury in piglets caused by weaning stress or diquat challenge [14, 15]. Emerging evidence also suggests the modulation of gut microbiota acts as a crucial mechanism for RSV to prevent atherosclerosis and high-fat diet-induced obesity [16, 17]. However, it is noteworthy that RSV has poor aqueous solubility and undergoes rapid phase-II conjugative metabolism in the intestine and liver, which gives it low bioavailability and may, in turn, limits its biological potency and application *in vivo* [18]. In this scenario, RSV derivatives with better bioavailability profiles, such as pterostilbene (PT), attract considerable attention from researchers.

PT is a naturally occurring methoxylated analog of RSV, sharing similar biological functions to those of RSV. Owing to the presence of methoxy groups, PT exhibits superior lipophilicity and metabolic stability to RSV, contributing to better cellular uptake and biological activities [18, 19]. Previous studies indicate that PT exerts a more potent inhibition than RSV in colon carcinogenesis and cervical cancer [20, 21]. However, the available information regarding the extensive comparison of RSV and PT *in vivo* is still scant. Also, there remains a crucial gap in understanding the protective effects of PT on intestinal oxidative stress and subsequent intestinal dysfunction. Therefore, the present study compared the *in vivo* protective potential of RSV and PT in intestinal redox status and gut microbiota using a porcine model of naturally occurring IUGR. Given the omnivorous nature and the similarities in intestinal anatomy and physiology between humans and pigs [22], this study may offer a potential therapeutic possibility for the intestinal syndrome in humans with IUGR.

## Materials and methods

### Animals and experimental design

A total of 72 healthy pregnant multiparous sows (Landrace × Yorkshire) with similar expected farrowing dates (less than 3 d) and parity (second or third) were preselected during gestation. At farrowing, the sows that had 11–13 live-born piglets and met the selection criteria for IUGR were chosen. Piglets were defined as intrauterine growth-retarded (IUGR) piglets when their birth weight were 2 standard deviations below the average birth weight of the total population, while those have a birth weight within 0.5 standard deviations of the average birth weight of the total population were deemed to be the normal body weight (NBW) piglets [4, 10]. After parturition, the body weight and sex of the newborn piglets (Duroc × [Landrace × Yorkshire]) were recorded within 3 h, and both NBW (1.56 ± 0.07 kg) and IUGR (0.90 ± 0.06 kg) piglets were kept with their dams until weaning. To avoid competition between siblings, the excess piglets were removed appropriately with each litter having nine piglets (one IUGR piglets per litter). At weaning (21 ± 1.49 days of age), the body weights of piglets were carefully recorded, and the candidates would be excluded from the study if they were unable to meet the selection criteria. Then, 18 NBW (6.81 ± 0.10 kg) and 54 IUGR (4.91 ± 0.06 kg) piglets from 54 litters were remained and moved to the weaner unit. The NBW piglets received a basal diet for 14 d (NBW group), while the IUGR piglets were assigned to one of three groups according to their body weight at weaning and received a basal diet (IUGR group), a basal diet with supplementation of 300 mg/kg RSV (RSV, purity ≥99%; CAS No: 501–36-0; BOC Sciences, Shirley, NY, USA; IUGR+RSV group), or a basal diet with supplementation of 300 mg/kg PT (PT, purity ≥99%; CAS No: 537–42-8; BOC Sciences; IUGR+PT group) for the same period, respectively. Each group consisted of 6 replicates (pens), with 3 piglets per replicate. The added level of RSV used in this study was determined according to previous publications [14, 23], in which supplementation with 300 mg/kg RSV in the diet improved the intestinal immune response, redox status, and barrier function of weanling piglets. An equivalent dosage of PT was chosen for IUGR+PT piglets to compare the protective potential of RSV and PT in the intestinal injury of IUGR piglets. The basal diet (Table S1) used in this study meets the NRC requirement (2012). All piglets received water and diet ad libitum during the trial period. The body weight and feed take of the piglets were recorded at d 21 and 35 of age on a pen basis to calculate the average daily gain (ADG), average daily feed intake (ADFI), and feed conversion ratio (FCR).

### Sample collection

At 35 days of age, all piglets were weighed, and one piglet having the nearest average weight of its replicate was selected for sampling. The blood samples were withdrawn into heparin-coated tubes from the precaval vein. The plasma samples were separated by centrifugation at 3,500×*g* for 15 min at 4 °C and then stored at − 80 °C until analysis. After blood sampling, the piglets were anaesthetised via electrical stunning and sacrificed by exsanguination. Subsequently, the abdominal cavity was opened and the jejunal segment was harvested immediately according to a previous study [24]. Approximately 1 cm segments in the middle of each jejunal samples were cut, flushed with ice-cold phosphate-buffered saline, and fixed in 4% paraformaldehyde solution for histomorphology examination. The jejunal mucosa was scraped from the rest of the jejunal tissues, frozen in liquid nitrogen, and stored at − 80 °C until further analysis. Finally, the cecal digesta was collected directly into sterile Eppendorf tubes and snap-frozen in liquid nitrogen before storage at − 80 °C for microbiota analysis.

### Measurement of circulating diamine oxidase (DAO) and D-lactate

Plasma DAO activity was determined using a colorimetric assay kit (#A008–1-1; Nanjing Jiancheng Bioengineering Institute, Nanjing, Jiangsu, China) according to the method of Hosoda et al. [25]. Briefly, nicotinamide adenine dinucleotide was quantitatively oxidized in the presence of glutamate dehydrogenase and α-ketoglutarate and detected at 340 nm, which in proportion to the amount of ammonia produced by DAO-catalyzed degradation of cadaverine. Plasma D-lactate was measured by kinetic spectrophotometric assay, using the D-lactate Colormetric Assay Kit MAK058 from Sigma (St. Louis, MO., USA). In this method, D-Lactate was specifically oxidized by D-Lactate hydrogenase and generated a proportional colorimetric product measured at 450 nm. All absorbance levels were detected using an ultraviolet-visible spectrophotometer (Tongfang Inc., Shanghai, China).

### Hematoxylin and eosin staining

After fixing in paraformaldehyde solution overnight, the jejunal samples were embedded and stained based on the method of Luna [26]. Briefly, the jejunum tissues were dehydrated in serial dilutions of alcohol, cleared in xylene, and embedded in paraffin in a vacuum. Then, the paraffin specimens were cut into 5-μm sections and subjected to a deparaffinization and graded rehydration procedure. Finally, the sections were stained with hematoxylin buffer and eosin buffer and viewed under a light microscope (Nikon ECLIPSE 80i, Tokyo, Japan). For quantitative analysis, 15 well-oriented and full-length crypt-villus units per jejunal sample were selected to determine the villus height (VH) and crypt depth (CD) using Image Pro Plus 6.0 software.

### Measurement of jejunal apoptosis

The apoptotic index of the jejunum was determined using a terminal deoxynucleotidyl transferase-mediated deoxyuridine triphosphate nick end labelling (TUNEL) BrightRed Apoptosis Detection Kit (#A113; Vazyme Biotech, Nanjing, Jiangsu, China) in accordance with the manufacturer’s guidance. After deparaffinization and aquation, the jejunal specimens were dipped into proteinase K (20 μg/mL) for 20 min at room temperature, followed by incubation with the TUNEL mixture (deoxynucleotidyl transferase enzyme and BrightRed Labeling Mix) at 37 °C for 1 h in a dark and humidified chamber. Subsequently, the slices were counterstained with the 4,6-diamidino-2-phenylindole solution (#P0131; Beyotime Institute of Biotechnology, Haimen, Jiangsu, China) and the images were captured using a fluorescence microscope (Nikon Eclipse C1; Nikon, Tokyo, Japan). The percentage of TUNEL-positive cells in the jejunum was quantified from 15 well-oriented villi each section by an independent and blinded observer who was not aware of the treatment procedures, using Image Pro Plus 6.0 software.

### Antioxidative capacity analysis

The jejunal mucosa tissues were homogenized in saline solution (1:4, weight:volume) and centrifuged at 3,500 r/min for 10 min. The supernatants were then diluted into the optimal content for detecting the activities of total superoxide dismutase (SOD; #A001–1), glutathione reductase (GR; #A062–1) and glutathione peroxidase (GPx; #A005–1), and the concentration of malondialdehyde (MDA; #A003–1) using the reagent kits (Nanjing Jiancheng Bioengineering Institute, Nanjing, Jiangsu, China). SOD activity was measured at 550 nm according to the method of Sun et al. [27], which depended on the inhibition of hydroxylamine oxidation that was directly proportional to the amount of SOD in the examined samples. Using the method of Saydam et al. [28], we assayed GR activity. This method was based on the oxidation of reduced nicotinamide adenine dinucleotide phosphate (NADPH) in the presence of GR and GSSG and the changes in absorbance at 340 nm were observed. A glutathione reduction reaction was used to evaluate the GPx activity, which was monitored at 412 nm [28]. MDA content in the tested samples was determined by thiobarbituric acid chromometry at 535 nm [29].

The activities of glutathione S-transferase (GST; #GST-2-W) and the concentrations of reduced glutathione (GSH; #GSH-2-W) and oxidized glutathione (GSSG; #GSSG-2-W) in the jejunal mucosa, were determined using commercial kits (Cominbio, Suzhou, Jiangsu, China). After homogenization of jejunum tissues in 0.1 M potassium phosphate buffer (pH = 7.4; 1:9, weight:volume) and centrifugation at 8,000 *g* for 10 min at 4 °C, the supernatants were obtained to detect GST activity by the procedure of Saydam et al. [28] using chlorodinitrobenzene as substrate. The formation of GSH-1-chloro-2,4-dinitrobenzene conjugate was monitored by the changes in absorbance at 340 nm. In addition, the jejunal tissues were homogenized with 5% metaphosphoric acid solution (1:9, weight:volume) and centrifugated at 8,000 *g* for 10 min at 4 °C for determination of GSH and GSSG levels according to the method of Teare et al. [30]. A chromophoric product, 2-nitro-5-thiobenzoic acid (TNB) resulting from the reaction of 5,5′-dithiobis-(2-nitrobenzoic acid) with GSH, was measured at 412 nm to determine the GSH content. GSSG was reduced to GSH in the presence of GR and NADPH. The rate of TNB formation was proportional to the total glutathione (the sum of GSH and GSSG, T-GSH) present and monitored at 412 nm. The GSSG concentration in the samples was calculated as the difference between T-GSH and GSH levels. All absorbance levels were detected using an ultraviolet-visible spectrophotometer (Tongfang Inc).

### Total RNA isolation and Quantitative Real-Time PCR (qRT-PCR) analysis

The RNAiso Plus Reagent (#9108; TaKaRa Biotechnology, Dalian, Liaoning, China) was used to isolate the total RNA of the jejunal mucosa. The quality and concentration of extracted RNA were detected using NanoDrop 1,000 (Thermo Fischer Scientific, Waltham, MA, USA), and the RNA integrity was evaluated with 2.0% agarose gel electrophoresis. Subsequently, the complementary DNA was produced using a PrimeScript™ RT Master Mix kit (#RR036A; TaKaRa Biotechnology). The qRT-PCR assay was conducted to determine the mRNA expression levels of *occludin* (*OCLN*), * claudin-1* (*CLDN-1*), *zonula occludens-1* (*ZO-1*), *NAD(P)H quinone dehydrogenase 1* (*NQO1*), *heme oxygenase 1* (*HO1*), *superoxide dismutase 1* (*SOD1*), *superoxide dismutase 2* (*SOD2*), and *beta-actin* (*ACTB*) using a TB Green® Premix Ex TaqTM kit (#RR420A; TaKaRa Biotechnology) with the QuantStudio 5 Real-time PCR System (Applied Biosystems, Life Technologies, CA, USA). The relative expression levels of the target genes were calculated using a quantification approach (2^-ΔΔCt^ method) and normalized against the reference gene (*ACTB*) expression level. A complete list of qRT-PCR primers is presented in Table S2.

### Protein extraction and western blot assays

Total protein isolation from the jejunal mucosa was carried out with a radioimmunoprecipitation assay lysis buffer containing protease inhibitor cocktail (#P0013B and #ST505; Beyotime Institute of Biotechnology, Jiangsu, China). The nuclear protein in the jejunal mucosa was prepared using a Nuclear Protein Extraction Kit (#P0027; Beyotime Institute of Biotechnology, Jiangsu, China). The concentrations of total cellular protein and nuclear protein in the jejunal mucosa were measured by the bicinchoninic acid protein assay kit (#P0010; Beyotime Institute of Biotechnology, Jiangsu, China). Thereafter, equal quantities of protein were resolved by SDS-PAGE gel and then transferred onto polyvinylidene difluoride membranes. After that, the membranes were incubated with blocking buffer (5% bovine serum albumin in Tris-buffered saline containing 1% Tween 20) for 1 h at room temperature and probed with the primary antibody against OCLN (#27260–1-AP; Proteintech, Chicago, IL, USA), CLDN-1 (#ab129119; Abcam, Cambridge, MA, USA), ZO-1 (#21773–1-AP; Proteintech), nuclear factor erythroid-2-related factor 2 (Nrf2; #ab92946; Abcam), Kelchlike ECH-associated protein-1 (Keap1; #10503–2-AP; Proteintech), NQO1 (#11451–1-AP; Proteintech), HO1 (#27282–1-AP; Proteintech), SOD1 (#10269–1-AP; Proteintech), SOD2 (#66474–1-Ig; Proteintech), Histone H3 (#17168–1-AP; Proteintech) and ACTB (#66009–10-Ig; Proteintech) overnight at 4 °C. Then, the membranes were washed by Tris-buffered saline with 0.05% Tween-20 and incubated with a suitable secondary antibody for 1 h at room temperature. Finally, these target bands were visualised using the Tanon 5200 Multi-Imaging System (Tanon Science & Technology, Inc., Shanghai, China) and analysed by Image Pro Plus 6.0 software.

### Cecal microbial community analysis by 16S rDNA gene sequencing

Total bacterial DNA was extracted from cecal digesta samples using the TIANamp Stool DNA Kit (#DP328, Tiangen Biotech, Beijing, China) according to the manufacturer’s guidance. The V3-V4 region of the bacteria 16S rDNA genes was amplified by PCR procedure with the specific primers (338F: 5′-ACTCCTACGGGAGGCAGCAG-3′ and 806R: 5′-GGACTACCVGGGTATCTAAT-3′) [31]. After checking the quality on a 2% agarose gel electrophoresis, the amplicons were purified using the AxyPrep DNA Gel Extraction Kit (#AP-GX-250, Axygen Biosciences, Union City, CA), pooled in equimolar, and sequenced pair end (2 × 300 bp) on an Illumina Hiseq platform (Huada Gene Institute, Beijing, China) according to the standard protocols.

After sequencing, the raw fastq files were preprocessed to remove the adapter pollution and the reads of low quality. The paired-end clean reads were merged to tags using FLASH (V 1.2.11). Subsequently, the clean tags were assigned into operational taxonomic units (OTUs) with a threshold of 97% identity by UPARSE (V 7.0.1090), and the chimeric sequences were identified and eliminated using UCHIME (V 4.2.40). Species annotation analysis was performed by an RDP Classifier (V 2.2) based on the Greengene database (V 201305) with a confidence threshold of 80%. The rarefaction curve, species accumulation curve, Shannon, Simpson, and Chao1 indices were calculated using RStudio software (V 3.5.3) with the vegan package. To estimate the beta diversity, principal coordinates analysis (PCoA) based on Bray-Curtis distance and the analysis of similarities (ANOSIM) were conducted to compare the differences between the treatments using RStudio software (V 3.5.3).

### Measurement of short-chain fatty acid in cecal digesta

The concentrations of cecal short-chain fatty acid, including acetate, propionate, and butyrate, were quantified using gas chromatography (GC-2014, Shimadzu, Kyoto, Japan) according to the method reported by Yu et al. [32].

### Statistical analysis

The SPSS for Windows version 22.0 statistical package (SPSS Inc., Chicago, IL, USA) was performed for statistical analysis. Dataset normality was determined by the Shapiro-Wilk test. When appropriate, one-way ANOVA and Tukey’s post hoc test were used to assess the significant difference among groups. Otherwise, the statistical significance of differences was analysed by the Kruskal–Wallis non-parametric test followed by pairwise differences in rank sums. The results were expressed as means with their standard errors. A *P*-value smaller than 0.05 was identified as statistically significant.

## Results

### Growth performance

In comparison with the NBW group, the IUGR piglets had lower initial and final body weight, ADG and ADFI (Table 1) during the 14 d after weaning (*P* < 0.05). Neither RSV nor PT supplementation significantly affected the growth performance of IUGR piglets (*P* > 0.05). There were no remarkable differences in initial and final body weight, ADG, ADFI, and FCR between the two supplemented groups (*P* > 0.05).

**Table 1:** Effect of dietary RSV and PT supplementation on the growth performance of IUGR piglets^1^

Items^2^	NBW	IUGR	IUGR+RSV	IUGR+PT	*P*-value
Initial BW, kg	6.81 ± 0.10^a^	4.94 ± 0.11^b^	4.89 ± 0.15^b^	4.90 ± 0.06^b^	< 0.001
Final BW, kg	9.61 ± 0.13^a^	6.84 ± 0.11^b^	6.97 ± 0.16^b^	7.14 ± 0.15^b^	< 0.001
ADG, g/d	200.20 ± 16.56^a^	136.11 ± 13.54^b^	149.01 ± 14.78^ab^	159.92 ± 8.35^ab^	0.020
ADFI, g/d	283.73 ± 22.22^a^	205.75 ± 19.37^b^	213.29 ± 16.95^ab^	228.37 ± 13.25^ab^	0.028
FCR, g/g	1.42 ± 0.02	1.52 ± 0.04	1.45 ± 0.05	1.43 ± 0.03	0.224

^a,b^Mean values with unlike superscript letters were significantly different (*P* < 0.05)

^1^There are no antibiotics in the basal diet. No piglets died during the trial period. The results were presented as means with their standard errors (*n* = 6). One-way ANOVA and Tukey’s post hoc test was conducted to determine the significant difference among the groups

^2^*ADG* average daily gain, *ADFI* average daily feed intake, *FCR* feed conversion ratio, *NBW* normal birth weight, *IUGR* intrauterine growth retardation, *RSV* resveratrol, *PT* pterostilbene

### Jejunal histomorphology and cell apoptosis

**Fig. 1:**
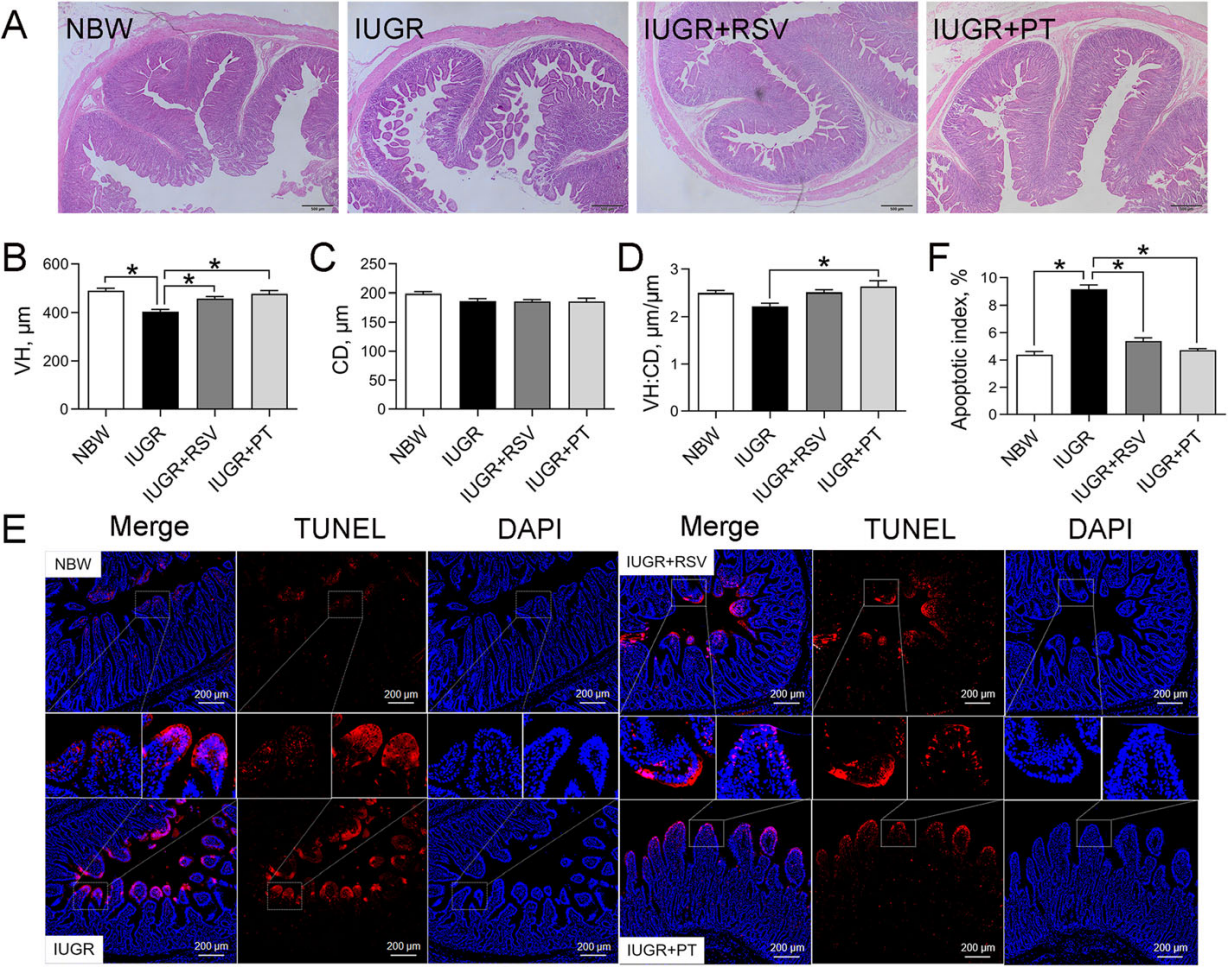
Effects of RSV and PT on jejunal morphology (**a-d**) and apoptosis (**e** and **f**) of IUGR piglets. Data were presented as means with standard errors (*n* = 6). One-way ANOVA and Tukey’s post hoc test was conducted to determine the significant difference among the groups. *Significantly different (*P* < 0.05) between groups. NBW, normal birth weight; IUGR, intrauterine growth retardation; RSV, resveratrol; PT, pterostilbene; VH, villus height; CD, crypt depth

As revealed by Fig. 1a, the jejunal villi of IUGR piglets failed to develop properly, as shown by the broken and shortened villi. In agreement with the histological observations, IUGR significantly reduced jejunal VH (Fig. 1b) and increased the number of jejunal TUNEL-positive cells (Fig. 1e and f), indicating a prominent jejunal injury of IUGR piglets (*P* < 0.05). In contrast, both RSV and PT administration effectively increased jejunal VH and reduced jejunal apoptotic index (*P* < 0.05). Moreover, a PT-supplemented diet also significantly increased the jejunal VH/CD ratio (Fig. 1d) of IUGR piglets (*P* < 0.05). The VH, CD (Fig. 1c), VH/CD ratio, and apoptotic index in the jejunum were similar between the IUGR+RSV and IUGR+PT groups (*P* > 0.05).

### Intestinal permeability and jejunal tight junction proteins levels

**Fig. 2:**
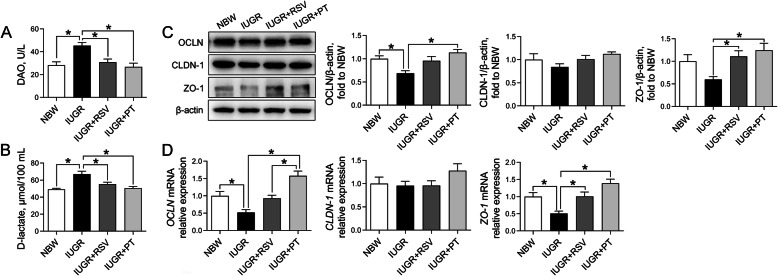
Effects of RSV and PT on the intestinal barrier function of IUGR piglets. **a** Plasma DAO activity; **b** Plasma D-lactate concentration; **c** The protein levels of jejunal OCLN, CLDN-1, and ZO-1; **d** The mRNA abundance of jejunal *OCLN*, *CLDN-1*, and *ZO-1*. Data were presented as means with standard errors (*n* = 6). One-way ANOVA and Tukey’s post hoc test was conducted to determine the significant difference among the groups. *Significantly different (*P* < 0.05) between groups. NBW, normal birth weight; IUGR, intrauterine growth retardation; RSV, resveratrol; PT, pterostilbene; DAO, diamine oxidase; OCLN, occludin; CLDN-1, claudin-1; ZO-1, zonula occludens-1

Compared with the NBW group, plasma DAO activity (Fig. 2a) and D-lactate concentration (Fig. 2b) were both significantly increased in the IUGR group, whereas these adverse alterations were totally reversed by both RSV- and PT-supplemented diets (*P* < 0.05). In addition, western blot analysis revealed that the IUGR piglets showed decreased protein levels of jejunal OCLN (Fig. 2c) compared with the NBW piglets, which was further verified by the qRT-PCR analysis (Fig. 2d; *P* < 0.05). Consistently, IUGR downregulated the gene expression of *ZO-1* in the jejunum compared with the NBW controls (*P* < 0.05). Conversely, both RSV and PT administration to IUGR piglets increased jejunal ZO-1 expression at transcriptional and translational levels compared with the IUGR subjects (*P* < 0.05). Moreover, PT markedly upregulated the mRNA and protein levels of jejunal OCLN of IUGR piglets and induced a higher *OCLN* mRNA abundance of IUGR+PT piglets than that in the IUGR-RSV group (*P* < 0.05). No significant differences were observed in jejunal *CLDN-1* mRNA or its protein levels among groups (*P* > 0.05).

### Jejunal redox status

**Fig. 3:**
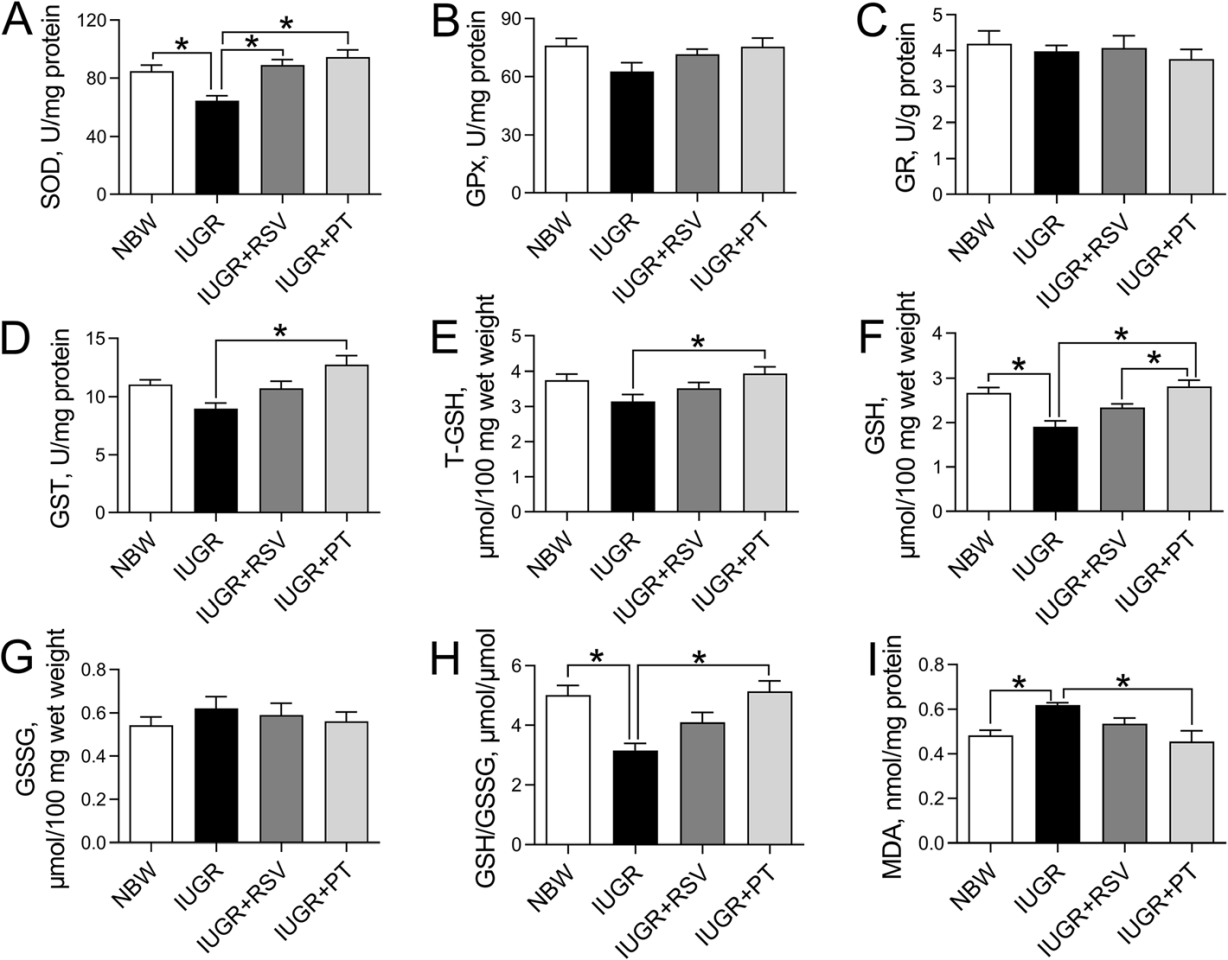
Effects of RSV and PT on jejunal redox status of IUGR piglets. **a-d** The activities of SOD, GPx, GR and GST of jejunum; **e-g** The concentrations of T-GSH, GSH and GSSG of jejunum; **h** Jejunal GSH/GSSG ratio; **i** Jejunal MDA level. Data were presented as means with standard errors (*n* = 6). One-way ANOVA and Tukey’s post hoc test was conducted to determine the significant difference among the groups. *Significantly different (*P* < 0.05) between groups. NBW, normal birth weight; IUGR, intrauterine growth retardation; RSV, resveratrol; PT, pterostilbene; SOD, total superoxide dismutase; GPx, glutathione peroxidase; GR, glutathione reductase; GST, glutathione S-transferase; T-GSH, total glutathione; GSH, reduced glutathione; GSSG, oxidative glutathione; MDA, malondialdehyde

The IUGR piglets displayed decreased SOD activity (Fig. 3a), GSH content (Fig. 3f), and GSH/GSGG ratio (Fig. 3h), and increased MDA concentration (Fig. 3i) in the jejunum when compared with the normal controls (*P* < 0.05). Nevertheless, the decreased jejunal SOD activity in the IUGR group was reversed to normal values by both RSV and PT treatment (*P* < 0.05). PT also obviously increased GST activity (Fig. 3d), the concentrations of T-GSH (Fig. 3e) and GSH, and GSH/GSSG ratio and inhibited the excessive accumulation of MDA in the jejunum of IUGR piglets (*P* < 0.05). Furthermore, we observed a higher jejunal GSH level in the IUGR+PT group when compared with the IUGR+RSV group (*P* < 0.05). No significant alterations were found in jejunal GPx (Fig. 3b), GR (Fig. 3c), or GSSG levels (Fig. 3g) among the piglets in the four treatments (*P* > 0.05).

### The expression of Nrf2 and its downstream target molecules in the jejunum

**Fig. 4:**
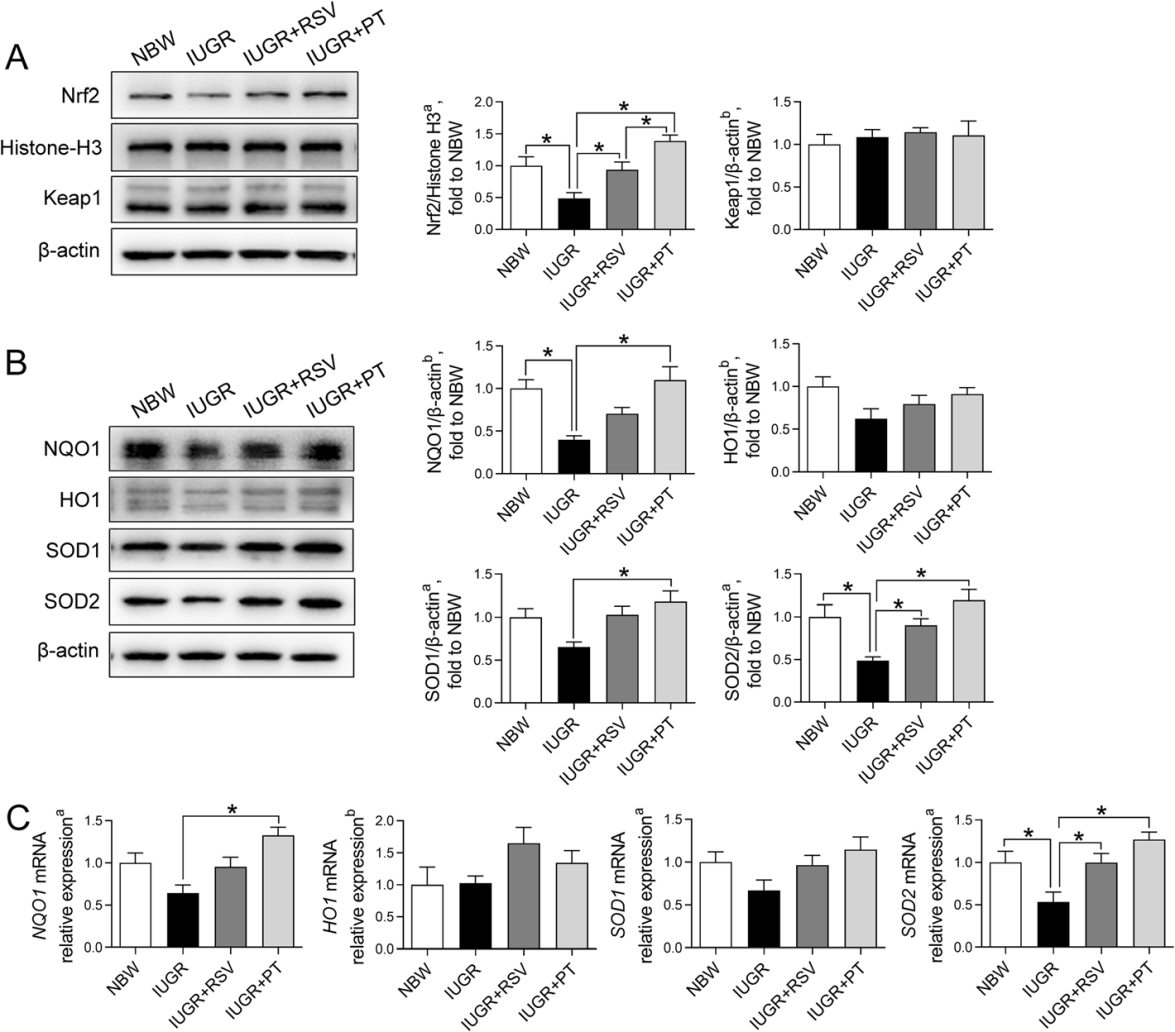
Effects of RSV and PT on the Nrf2 pathway in the jejunum of IUGR piglets. **a** The protein levels of nuclear Nrf2 and cellular Keap1 in the jejunum; **b** The protein levels of NQO1, HO1, SOD1 and SOD2 in the jejunum; **c** The mRNA abundance of *NQO1*, *HO1*, *SOD1* and *SOD2* in the jejunum. Data were presented as means with standard errors (*n* = 6). ^a^One-way ANOVA and Tukey’s post hoc test was conducted to determine the significant difference among the groups. ^b^Kruskal–Wallis non-parametric test followed by pairwise differences in rank sums was conducted to determine the significant difference among the groups. *Significantly different (*P* < 0.05) between groups. NBW, normal birth weight; IUGR, intrauterine growth retardation; RSV, resveratrol; PT, pterostilbene; Nrf2, nuclear factor erythroid 2-related factor 2; Keap1, Kelchlike ECH-associated protein-1; NQO1, NAD(P)H quinone dehydrogenase 1; HO1, heme oxygenase 1; SOD1, superoxide dismutase 1; SOD2, superoxide dismutase 2

Compared with the normal controls, the nuclear translocation level of jejunal Nrf2 (Fig. 4a) of IUGR piglets was markedly decreased (*P* < 0.05), but the protein level of Keap1, a repressor of Nrf2, was not altered by IUGR (*P* > 0.05). In parallel with the lower nuclear Nrf2 protein level, IUGR downregulated jejunal protein levels of NQO1 and SOD2 (Fig. 4b), two important downstream targets of Nrf2 (*P* < 0.05). Moreover, the lower jejunal *SOD2* mRNA abundance (Fig. 4c) was also found in the IUGR piglets (*P* < 0.05). In contrast, both RSV and PT supplementation dramatically stimulated the nuclear Nrf2 accumulation in the jejunum of IUGR piglets (*P* < 0.05); PT exhibited a more remarkable effect than RSV in this process (*P* < 0.05). Meanwhile, piglets in two supplemented groups displayed higher mRNA and protein levels of jejunal SOD2 relative to the IUGR piglets (*P* < 0.05). PT treatment to IUGR piglets also increased the mRNA and protein levels of NQO1 and the protein level of SOD1 in the jejunum, compared with those that received a control diet (*P* < 0.05).

### Cecal microbiota composition

**Fig. 5:**
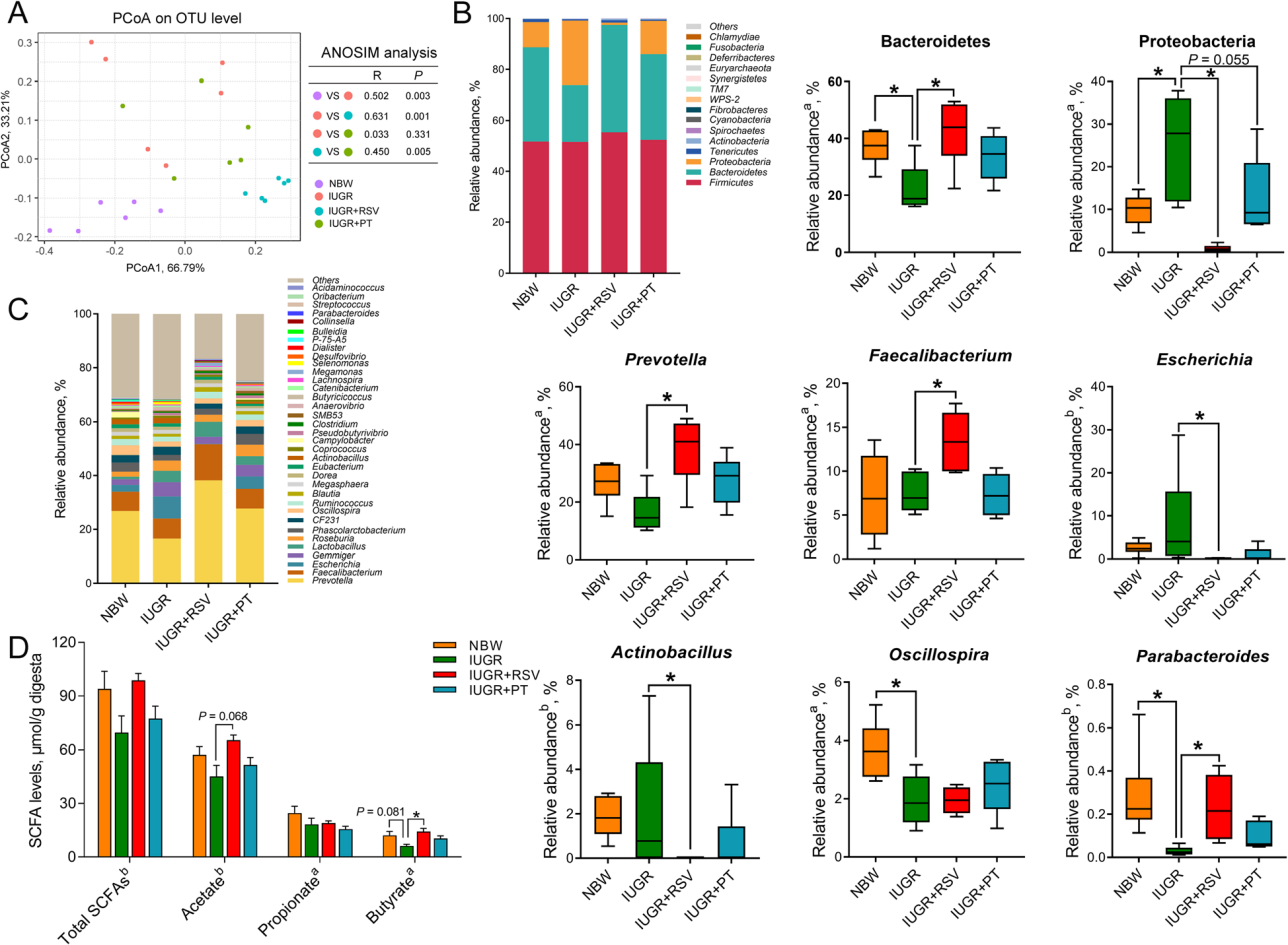
Effects of RSV and PT on the cecal microbiota structure and short-chain fatty acid concentrations of IUGR piglets. **a** PCoA based on the Bray-Curtis distance and ANOSIM; **b** Phylum level taxonomic distributions of the microbial communities of cecum; **c** Relative abundance of the top 35 microbial genera of cecum; **d** Cecal short-chain fatty acid concentrations. Data were presented as means with standard errors (*n* = 6). ^a^One-way ANOVA and Tukey’s post hoc test was conducted to determine the significant difference among the groups. ^b^Kruskal–Wallis non-parametric test followed by pairwise differences in rank sums was conducted to determine the significant difference among the groups.*Significantly different (*P* < 0.05) between groups. NBW, normal birth weight; IUGR, intrauterine growth retardation; RSV, resveratrol; PT, pterostilbene; PCoA, Principal coordinate analysis; ANOSIM, analysis of similarities

To investigate the impact of RSV and PT on the gut microbial environment, we profiled the cecal bacterial community composition of each treatment using 16S rDNA gene sequencing. The rarefaction analysis and species accumulation curve (Fig. S1) illustrated that high diversity of the microbial community and sequencing depth have been achieved in this work. After sequencing, a total of 730 OTUs were identified from all samples, and 493 shared OTUs out of the total OTUs overlapped among the four groups (Fig. S2A). The alpha diversity reflects species richness (Chao1 index) and species diversity (Simpson and Shannon index). In this study, IUGR induced a lower Shannon index (Fig. S2B; *P* < 0.05) and tended to decrease the Chao1 index (*P* = 0.054) in comparison with the NBW piglets, which were not improved by RSV or PT (*P* > 0.05). We also determined the beta diversity of the cecal microbiota by the PCoA analysis (Fig. 5a), which unveiled that there was an apparent cluster of the cecal microbiota structures among the four groups. The results of ANOSIM analysis further suggested remarkable separation between the cecal microbiota of NBW and IUGR (*R* = 0.502, *P* = 0.003), IUGR and IUGR+RSV (*R* = 0.631, *P* = 0.001), and IUGR+RSV and IUGR+PT (*R* = 0.450, *P* = 0.005) groups. However, the cecal bacterial communities in the IUGR and IUGR+PT groups were similar (*R* = 0.033, *P* = 0.331).

In order to further identify the changes in cecal microbiota composition, the dominant phyla and genera in all the groups were analysed. At the phylum level (Fig. 5b), Firmicutes, Bacteroidetes, and Proteobacteria predominantly constituted the cecal microbiota of piglets. Compared with the NBW group, IUGR decreased the relative abundance of Bacteroidetes and enhanced the relative abundance of Proteobacteria (*P* < 0.05). Instead, these alterations of IUGR piglets were reversed by an RSV diet (*P* < 0.05). Moreover, PT intervention also tended to reduce the relative abundance of Proteobacteria (*P* = 0.055). At the genus level, a total of 79 bacterial genera were annotated and the top 35 of which were depicted in Fig. 5c. Compared with the NBW controls, the relative abundance of the dominant taxa *Oscillospira* and *Parabacteroides* of IUGR piglets were decreased (*P* < 0.05). After 2-weeks RSV supplementation, the relative abundance of *Prevotella*, *Faecalibacterium*, and *Parabacteroides* were significantly increased, and the relative abundance of *Actinobacillus* and *Escherichia* were remarkably decreased (*P* < 0.05). These results collectively demonstrated that both RSV and PT modulated the structure of cecal microbes of IUGR piglet.

### Short-chain fatty acid levels in cecal digesta

As illustrated in Fig. 5d, IUGR tended to decrease the butyrate concentration of cecal digesta compared with the NBW group (*P* = 0.081), and no significant differences were observed in the concentrations of total short-chain fatty acid, acetate, or propionate between the NBW and the IUGR groups (*P* > 0.05). In contrast, RSV intervention significantly increased the butyrate concentration (*P* < 0.05) and tended to enhance the acetate concentration in cecal digesta (*P* = 0.068).

## Discussion

IUGR animals often exhibit growth lag in their early growth stage [5, 7, 33]. One of the predisposing factors is that IUGR impairs mucosal morphology and decreases the activities of digestive enzymes in the intestine, leading to the low efficiency of nutrient utilization [33]. In addition, the defective antioxidant system of the IUGR intestines may contribute to uncontrolled oxidative stress and subsequent local and systemic damage, thereby decreasing the growth performance of young animals [7]. Recently, researchers proposed that the changes in intestinal microbiota communities of IUGR pigs are associated with their poor growth outcomes [31, 34]. The present study demonstrated that both RSV and its derivative PT retained jejunal villus-crypt architecture, prevented jejunal excessive apoptosis, and maintained the intestinal integrity of IUGR piglets. RSV and PT treatment also ameliorated IUGR-associated jejunal oxidative stress and cecal microbiota dysbiosis of piglets. Notably, neither RSV nor PT improved the ADG, ADFI, and FCR of IUGR piglets, which is consistent with previous studies showing that RSV did not alter the growth performance of weanling and finishing pigs [35–37]. Nevertheless, Cao et al. [15] reported that dietary RSV supplementation enhanced the ADG and ADFI of piglets under acute oxidative stress. Our recent study also demonstrated that dietary PT supplementation efficiently prevented the weight loss of weanling piglets challenged by diquat injection [38]. Therefore, the effects of stilbenes on the growth performance may be probably related to the discrepancies in physiological conditions of pigs, and it needs more investigations.

Intestinal integrity plays a fundamental role in intestinal physiological functions, which can be evaluated by the circulating DAO activity and D-lactate concentration [39]. In this study, we found that plasma DAO activity and D-lactate level in the IUGR+RSV and IUGR+PT groups were significantly decreased, which may be closely associated with the upregulation of tight junction proteins. Intestinal tight junction complex acts as a structural and functional barrier against the paracellular flux of luminal substances comprising a variety of transmembrane proteins, such as OCLN, ZO, and CLDN family members [3]. After a 2-weeks feeding trial, both RSV and PT increased the jejunal ZO-1 expression at the transcriptional and translational levels and PT also enhanced the protein abundance of OCLN. In addition, the anti-apoptotic activities exerted by RSV and PT also had significant implications in this protection. El-Boghdady et al. [40] demonstrated that RSV can regulate the ratio of anti-apoptotic to pro-apoptotic proteins to alleviate paraquat-induced hepatic injury. Likewise, in a porcine model of oxidative stress, PT was found to prevent hepatic apoptosis in the mitochondria-dependent pathway [38]. Our results validated these findings and indicated that RSV and PT alleviated excessive apoptosis in the jejunum of IUGR piglets. The intestinal epithelium is one of the fastest proliferating and self-renewing tissues. The perturbation of proliferation-apoptosis homeostasis can hinder enterocyte turnover and further induce villi atrophy [41]. Thus, the repressed apoptosis in the RSV and PT groups further explains the restoration of the intestinal architecture of IUGR piglets. Collectively, RSV and its derivative PT may maintain the intestinal integrity of the IUGR piglets by increasing tight junction protein expression and inhibiting enterocyte apoptosis.

Oxidative stress has been recognized as a crucial mechanism in intestinal disorders of IUGR neonates [7, 10]. SOD is a crucial antioxidative enzyme responsible for the prominent defensive mechanism against superoxide radicals and cellular redox balance [42]. In this study, both RSV and PT administration enhanced jejunal SOD activity as well as the mRNA and protein levels of SOD2 in IUGR piglets, suggesting that the upregulated expression and activities of antioxidant enzymes may be a crucial mechanism by which RSV and PT prevent the IUGR-associated intestinal injury. Interestingly, although SOD activity and expression were stimulated after stilbenes treatment, only PT effectively reversed the overproduction of jejunal MDA of IUGR piglets, which implies that supplementation with RSV at the present dosage appears to be insufficient to protect against the IUGR-associated oxidative stress in the jejunum. Similarly, Mikstacka et al. [43] also observed that PT rather than RSV effectively alleviated lipid peroxidation in human erythrocytes caused by hydrogen peroxide. The structural difference between RSV and PT may provide a possible reason for these findings. Because the substitution of the hydroxy groups with methoxy groups creates a higher lipophilicity and better cell membrane permeability of PT, which may help to increase the accessibility of PT into the enterocytes [44–48].

On the other hand, the potent potential of PT in the activation of Nrf2 pathway was also found in this study, which is consistent with a previous study suggesting that PT administration induced an approximately four-fold increase in Nrf2 protein levels in the colon compared with RSV at an equivalent dose [20]. Nrf2 is a master transcription factor regulating antioxidant response and maintaining cellular homeostasis. Upon activation, the Nrf2/Keap1 complex is dissociated, and Nrf2 is released from Keap1 and translocates into the nucleus, where it forms a heterodimer with its obligatory partner Maf and binds to the antioxidant response element to activate transcription of a battery of antioxidant and cytoprotective genes. It has been demonstrated that PT can form favorable interaction with the arginine triad residues of Keap1 kelch domain, which facilitates the dissociation of Nrf2/Keap1 complex [49]. Taken together, dietary administration of RSV and PT alleviates IUGR-associated intestinal oxidative stress of piglets, and this positive effect of PT was stronger than that of RSV.

Intestinal flora is the crucial determinant of intestinal development and immune system maturation. Previous studies revealed that low birth weight can disturb gut microbiota [31, 34], which was confirmed by the present study. Proteobacteria is one of the dominant phyla of gram-negative bacteria, consisting of various harmful pathogens, such as *Escherichia* and *Actinobacillus* [50, 51]. It is reported that a chronic prevalence of Proteobacteria is closely associated with an imbalanced microbial structure or intestinal diseases in humans and animals [52, 53]. After receiving RSV or PT, the cecal abundance of Proteobacteria of IUGR piglets was decreased and RSV also downregulated the abundance of *Escherichia* and *Actinobacillus*, indicating the potential of RSV and PT in suppressing the proliferation of harmful bacteria. Bacteroidetes is the most predominant anaerobe producing short-chain fatty acid in the gut, which can be modulated by RSV to maintain energy metabolism homeostasis in high-fructose or high-lipid models [17, 53]. In agreement with these studies, we found that the abundance of the phylum Bacteroidetes and its genera *Prevotella* and *Parabacteroides* in the cecum was enhanced by RSV. *Faecalibacterium* is one of the main butyrate producers, which provides energy for the gut epithelial cells and confers anti-inflammatory properties [54]. Zhou et al. [55] illustrated that *Faecalibacterium prausnitzii* mitigates colorectal colitis through production of butyrate and modulation of the Th17/Treg balance. Here, we observed apparent increases in the prevalence of *Faecalibacterium* and butyrate levels in the cecum after RSV treatment, suggesting that RSV may avail the intestinal health by regulating potential probiotics and their metabolites in the gut.

It is worth noting that the ability of RSV to regulate gut microbiota was higher than that of PT, which seems in striking contrast to its insufficient antioxidative activity of IUGR piglets mentioned above. Actually, given the low bioavailability/high bioactivity paradox of RSV, growing evidence supports the hypothesis that RSV is possibly acting primarily through remodeling the gut microbiota [56, 57]. Although the exact mechanism has not been ascertained yet, it has been demonstrated that RSV significantly regulated the growth of certain gut microbiota *in vivo*, such as increasing the Bacteroidetes-to-Firmicutes ratios and the growth of *Bacteroides*, *Lactobacillus*, and *Bifidobacterium* [17, 53, 57]. Interestingly, some gut microbiota is also able to affect RSV’s metabolism and determine its fate and physiologic effects. Dihydroresveratrol, 3,4′-dihydroxy-trans-stilbene, and 3,4′-dihydroxybibenzyl have been identified as the main microbial-derived metabolites of RSV [58, 59], which implies that the reduction of the carbon-carbon double bond and cleavage of hydroxyl groups may be the crucial interactions between RSV and gut microbiota. In contrast, even though PT has a carbon-carbon double bond as that of RSV, no metabolites of PT with reduced double bond were identified yet [19]. Also, recent studies have revealed that the demethylation may be a potential microbial metabolic pathway of PT [19, 60]. Thus, we speculate the differences in microbial biotransformation between RSV and PT may provide a probable explanation for their different effects on gut microbiota. However, further investigations are required to validate.

## Conclusion

Both RSV and PT protect against the IUGR-associated intestinal morphological changes, increased intestinal permeability, redox imbalance, and gut dysbiosis of weanling piglets. PT shows more potency than RSV in alleviating oxidative stress and activating the Nrf2-associated antioxidant system in the jejunum of IUGR piglets. RSV is superior to PT in regulating the cecal microbiota of IUGR piglets.

## Supplementary Information

**Additional file 1: Table S1.** Composition and nutrient levels of the basal diet.

**Additional file 2: Table S2.** Primer sequences for quantitative real-time PCR.

**Additional file 3: Figure S1.** Rarefaction curve and species accumulation curve of cecal microbiota based on operational taxonomic units.

**Additional file 4: Figure S2.** Unique and shared OTUs analysis and the alpha diversity of the cecal microbiota.

## Data Availability

The datasets generated and/or analysed during this study are available from the corresponding author on reasonable request.

## Abbreviations

ACTB: Beta-actin
ANOSIM: The analysis of similarities
ADFI: Average daily feed intake
ADG: Average daily gain
CD: Crypt depth
CLDN-1: Claudin-1
DAO: Diamine oxidase
FCR: Feed conversion ratio
GPx: Glutathione peroxidase
GR: Glutathione reductase
GSH: Reduced glutathione
GSSG: Oxidized glutathione
GST: Glutathione S-transferase
HO1: Heme oxygenase 1
IUGR: Intrauterine growth retardation
Keap1: Kelchlike ECH-associated protein-1
MDA: Malondialdehyde
NADPH: Reduced nicotinamide adenine dinucleotide phosphate
NBW: Normal body weight
NQO1: NAD(P)H quinone dehydrogenase 1
Nrf2: Nuclear factor erythroid 2-related factor 2
OCLN: Occludin
OTUs: Operational taxonomic units
PCoA: Principal coordinates analysis
PT: Pterostilbene
qRT-PCR: Quantitative real-time PCR
RSV: Resveratrol
SOD: Superoxide dismutase
TNB: 2-nitro-5-thiobenzoic acid
TUNEL: Terminal deoxynucleotidyl transferase-mediated deoxyuridine triphosphate nick end labelling
T-GSH: Total glutathione
VH: Villus height
ZO-1: Zonula occludens-1
