# Supplementary material for: Resveratrol and its derivative pterostilbene ameliorate intestine injury in intrauterine growth-retarded weanling piglets by modulating redox status and gut microbiota
Source: J Anim Sci Biotechnol. 2021 Jun 10;12:70. doi: 10.1186/s40104-021-00589-9 (PMC8191009; doi:10.1186/s40104-021-00589-9)
Supplement: Supplementary file 1 — Additional file 1: Table S1. Composition and nutrient levels of the basal diet. [file 40104_2021_589_MOESM1_ESM.docx]

**Table S1.** Composition and nutrient levels of the basal diet (%, as-fed basis unless otherwise stated)

| Items | % | Nutrient levels^a^ | % |
| --- | --- | --- | --- |
| Maize | 62.78 | Digestible energy, Mcal/kg | 3.47 |
| Soybean meal | 15.00 | Metabolizable energy, Mcal/kg | 3.30 |
| Fermented soybean meal | 7.00 | Crude protein | 20.36 |
| Extruded soybean | 7.00 | Total lysine | 1.51 |
| Soy protein isolate | 1.30 | Total methionine | 0.46 |
| Soyabean oil | 2.00 | Total methionine + cystine | 0.86 |
| CaHPO_4_ | 1.80 | Total threonine | 0.94 |
| Limestone | 0.80 | Total tryptophan | 0.40 |
| Salt | 0.35 | Total histidine | 0.77 |
| L-lysine-HCl, 78% | 0.52 | Total isoleucine | 0.79 |
| L-methionine | 0.13 | Total valine | 1.20 |
| L-threonine | 0.15 | Total calcium | 0.82 |
| L-isoleucine | 0.10 | Total phosphorus | 0.65 |
| L-tryptophan | 0.01 | SID^c^ amino acids / Lysine | % |
| L-histidine | 0.01 | Methionine | 29.85 |
| Calcium propionate, 50% | 0.05 | Methionine + Cystine | 53.73 |
| Premix^b^ | 1.00 | Threonine | 59.70 |
| Total | 100.00 | Tryptophan | 26.12 |
|  |  | Histidine | 48.51 |
|  |  | Isoleucine | 52.24 |
|  |  | Valine | 77.61 |

^a^All nutrient levels were analyzed values, except digestible energy and metabolizable energy.

^b^Provide the following per kg complete diet: Vitamin A, 8,000 IU; Vitamin D_3_, 3,000 IU; Vitamin E, 20 IU; Vitamin K_3_, 3 mg; Vitamin B_1_, 2 mg; Vitamin B_2_, 5 mg; Vitamin B_6_, 7 mg; Vitamin B_12_, 0.02 mg; Niacin, 30 mg; Pantothenic acid, 15 mg; Folic acid, 0.3 mg; Biotin, 0.08 mg; Choline chloride, 500 mg; Fe (from ferrous sulfate), 110 mg; Cu (from copper sulfate), 7 mg; Mn (from manganese sulfate), 5 mg; Zn (from zinc sulfate), 110 mg; I (from calcium iodate), 0.3 mg; Se (from sodium selenite), 0.3 mg.

^c^SID, Standardized ileal digestible.
