# Supplementary material for: Resveratrol and its derivative pterostilbene ameliorate intestine injury in intrauterine growth-retarded weanling piglets by modulating redox status and gut microbiota
Source: J Anim Sci Biotechnol. 2021 Jun 10;12:70. doi: 10.1186/s40104-021-00589-9 (PMC8191009; doi:10.1186/s40104-021-00589-9)
Supplement: Supplementary file 2 — Additional file 2: Table S2. Primer sequences for quantitative real-time PCR. [file 40104_2021_589_MOESM2_ESM.docx]

**Table S2**. Primer sequences used for quantitative real-time PCR

| Gene name^1^ | Genbank^2^ | Sequence (5’→3’^3^) | Length |
| --- | --- | --- | --- |
| *OCLN* | NM_001163647.2 | CAGGTGCACCCTCCAGATTG | 167 |
|  |  | ATGTCGTTGCTGGGTGCATA |  |
| *CLDN-1* | XM_005670262.3 | CTAGTGATGAGGCAGATGAA | 250 |
|  |  | AGATAGGTCCGAAGCAGAT |  |
| *ZO-1* | XM_005659811.1 | GACCCGGCCAAGGTGTATAG | 75 |
|  |  | TGGCTGCTTCAAGACATGGT |  |
| *NQO1* | NM_001159613.1 | CATGGCGGTCAGAAAAGCAC | 135 |
|  |  | ATGGCATACAGGTCCGACAC |  |
| *HO1* | NM_001004027.1 | TGATGGCGTCCTTGTACCAC | 71 |
|  |  | GACCGGGTTCTCCTTGTTGT |  |
| *SOD1* | NM_001190422.1 | AAGGCCGTGTGTGTGCTGAA | 118 |
|  |  | GATCACCTTCAGCCAGTCCTTT |  |
| *SOD2* | NM_214127.2 | GGCCTACGTGAACAACCTGA | 126 |
|  |  | TGATTGATGTGGCCTCCACC |  |
| *ACTB* | XM_003124280.5 | TGGAACGGTGAAGGTGACAG | 176 |
|  |  | CTTTTGGGAAGGCAGGGACT |  |

^1^*ACTB*, Beta actin; *CLDN-1*, Claudin-1; *HO1*, Haem oxygenase 1; *NQO1*, NAD(P)H quinone dehydrogenase 1; *OCLN*, Occludin; *SOD1*, Superoxide dismutase 1; *SOD2*, Superoxide dismutase 2; *ZO-1*, Zonula occludens-1.

^2^GenBank Accession Number.

^3^Shown as the forward primer followed by the reverse primer.
