# Supplementary material for: Resveratrol and its derivative pterostilbene ameliorate intestine injury in intrauterine growth-retarded weanling piglets by modulating redox status and gut microbiota
Source: J Anim Sci Biotechnol. 2021 Jun 10;12:70. doi: 10.1186/s40104-021-00589-9 (PMC8191009; doi:10.1186/s40104-021-00589-9)
Supplement: Supplementary file 3 — Additional file 3: Figure S1. Rarefaction curve and species accumulation curve of cecal microbiota based on operational taxonomic units. [file 40104_2021_589_MOESM3_ESM.docx]

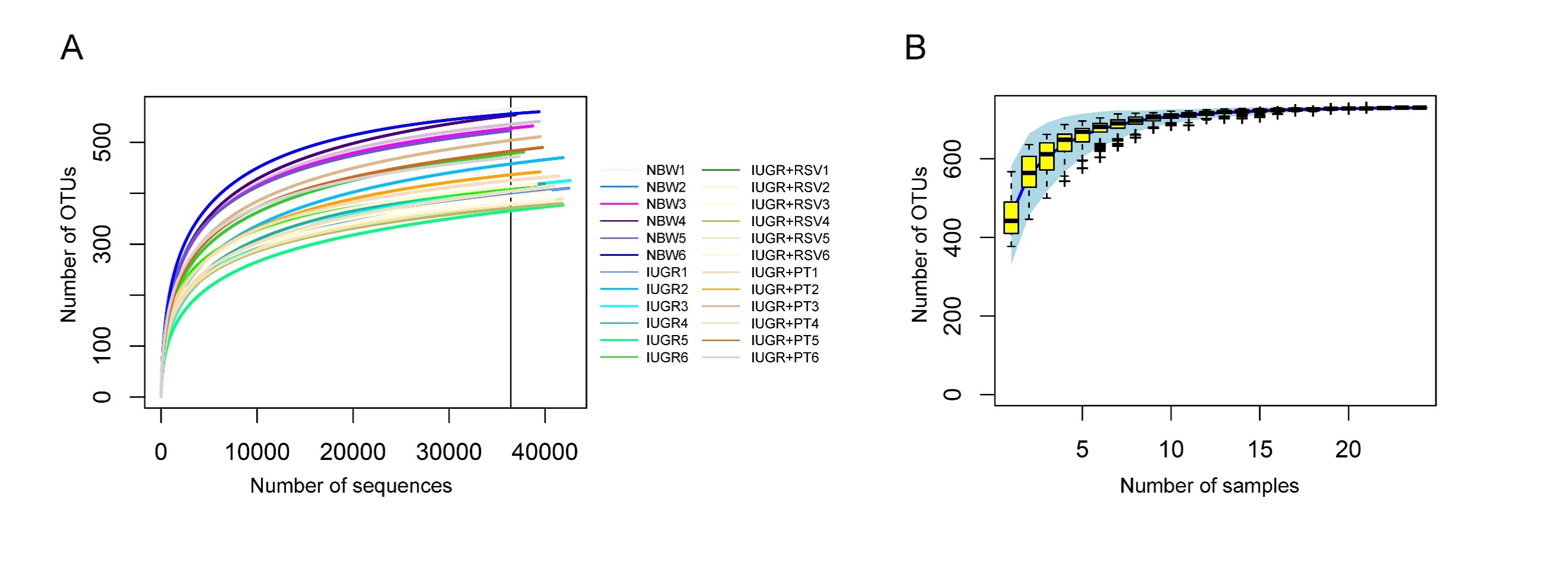


**Figure S1**. Rarefaction curve (A) and species accumulation curve (B) of the cecal microbiota based on operational taxonomic units (OTUs).
