# Supplementary figures and images for: Resveratrol and its derivative pterostilbene ameliorate intestine injury in intrauterine growth-retarded weanling piglets by modulating redox status and gut microbiota
Source: J Anim Sci Biotechnol. 2021 Jun 10;12:70. doi: 10.1186/s40104-021-00589-9 (PMC8191009; doi:10.1186/s40104-021-00589-9)

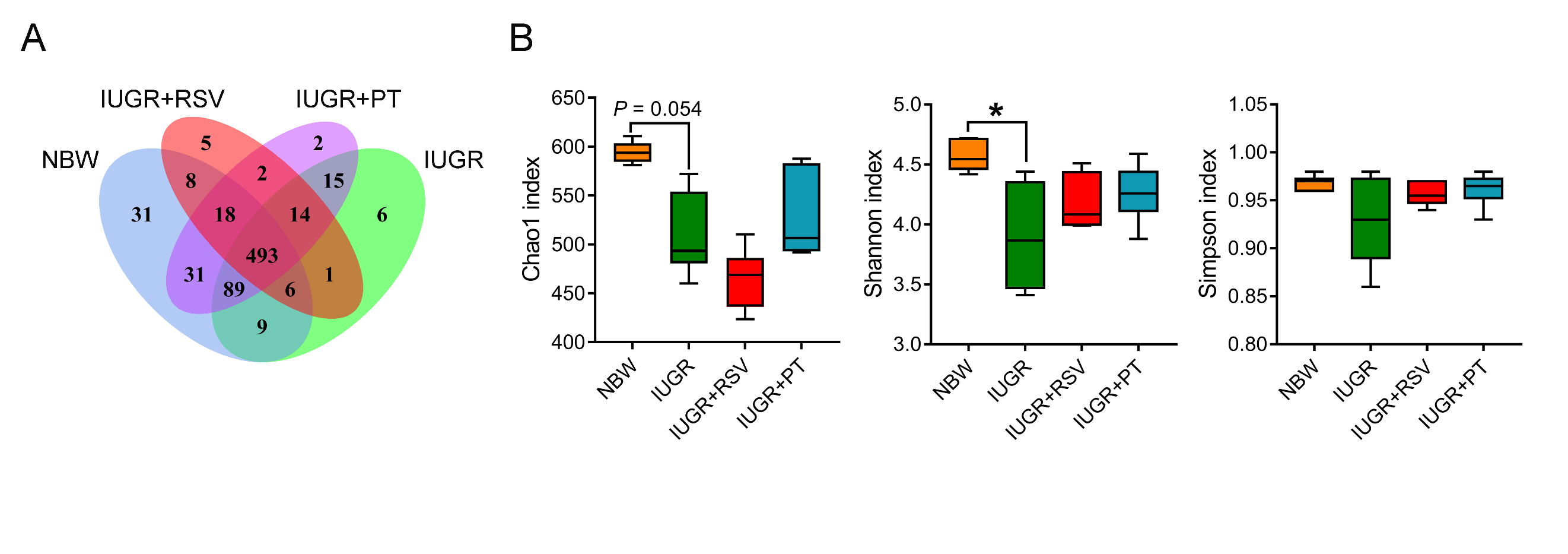


**Figure S2**. Unique and shared OTUs analysis (A) and the alpha diversity (B) of the cecal microbiota.

Supplement: Supplementary file 4 — Additional file 4: Figure S2. Unique and shared OTUs analysis and the alpha diversity of the cecal microbiota. [file 40104_2021_589_MOESM4_ESM.docx]
